# Supplementary material for: Estimating societal benefits from Nordic catchments: An integrative approach using a final ecosystem services framework
Source: PLoS One. 2021 Jun 1;16(6):e0252352. doi: 10.1371/journal.pone.0252352 (PMC8168860; doi:10.1371/journal.pone.0252352)
Supplement: S2 File — (DOCX) [file pone.0252352.s002.docx]

Supplement 2 – Overview of spatial datasets

This table shows spatial datasets used in TEV estimations. It shows per type of spatial dataset which FES are linked to it, as well as the data source. Links to the data source are supplied in the footnote.

| Dataset type | | Related FES | Sources | |  |
| --- | --- | --- | --- | --- | --- |
| Land cover | Crop production  Forestry  Game  Berries and mushrooms  Carbon sequestration  Downstream flood reduction  Recreational possibilities | | | CORINE land cover (Copernicus)^[[1]](#footnote-1)^  Norwegian Insititute for Bioeconomy Research^[[2]](#footnote-2)^  Miljøstyrelsen Denmark^[[3]](#footnote-3)^  Natural Resources Institute Finland^[[4]](#footnote-4)^  Finnish Agency for Rural Affairs  Swedish Environmental Protection Agency^[[5]](#footnote-5)^ | |
| River network | Downstream flood reduction  Recreational possibilities | | | Norwegian Water Resources and Energy Directorate  Aarhus University  Finnish Environment Institute^[[6]](#footnote-6)^  Länsstyrelsen Vattenkartan^[[7]](#footnote-7)^ | |
| Road network | Recreational possibilities | | | Felles kartdatabase^[[8]](#footnote-8)^  Miljøstyrelsen Denmark  National Land Survey of Finland^[[9]](#footnote-9)^  Trafikverket^[[10]](#footnote-10)^ | |
| Flood zones | Downstream flood reduction | | | Norwegian Water Resources and Energy Directorate  Kystdirektoratet^[[11]](#footnote-11)^  Finnish Environment Institute  Myndigheten för samhällsskydd och beredskap^[[12]](#footnote-12)^ | |
| Biomass growth | Forestry  Carbon sequestration | | | Norwegian Insititute for Bioeconomy Research  Natural Resources Institute Finland  Swedish Environmental Protection Agency | |

| Agricultural production | Crop production | Norwegian Insititute for Bioeconomy Research  Danmarks Miljøportal^[[13]](#footnote-13)^  Länsstyrelsen Uppsala / Västerbotten län^[[14]](#footnote-14)^  Agency for Rural Affairs^[[15]](#footnote-15)^ |
| --- | --- | --- |
| Population density | Game  Berries and mushrooms  Water for drinking and processing  Recreational possibilities | Statistics Norway^[[16]](#footnote-16)^  Worldpop^[[17]](#footnote-17)^ |
| Slope | Crop production  Livestock production  Downstream flood reduction | EU-DEM (Copernicus)^[[18]](#footnote-18)^ |
| Hydropower plant locations | Electricity production | Norges vassdrags- og energidirektorat^[[19]](#footnote-19)^ |
| Areas of peat production | Peat extraction | Oulu University^[[20]](#footnote-20)^ |

1. https://land.copernicus.eu/pan-european/corine-land-cover [↑](#footnote-ref-1)
2. https://kartkatalog.geonorge.no/ [↑](#footnote-ref-2)
3. Email from Fatemeh Hashemi (Aarhus University) [↑](#footnote-ref-3)
4. https://kartta.paikkatietoikkuna.fi/?lang=en [↑](#footnote-ref-4)
5. https://miljodataportalen.naturvardsverket.se/miljodataportalen/ [↑](#footnote-ref-5)
6. https://kartta.paikkatietoikkuna.fi/?lang=en [↑](#footnote-ref-6)
7. https://ext-geoportal.lansstyrelsen.se/ [↑](#footnote-ref-7)
8. https://kartkatalog.geonorge.no/ [↑](#footnote-ref-8)
9. https://kartta.paikkatietoikkuna.fi/?lang=en# [↑](#footnote-ref-9)
10. https://ext-geodatakatalog.lansstyrelsen.se/GeodataKatalogen/ [↑](#footnote-ref-10)
11. https://oversvommelse.kyst.dk/planperiode-2016-2021/plantrin-1/vandloebsoversvoemmelser/ [↑](#footnote-ref-11)
12. https://gisapp.msb.se/apps/oversvamningsportal/avancerade-kartor/oversvamningskartering.html [↑](#footnote-ref-12)
13. https://miljoeportal.dk/ [↑](#footnote-ref-13)
14. https://ext-geodatakatalog.lansstyrelsen.se/GeodataKatalogen/ [↑](#footnote-ref-14)
15. https://kartta.paikkatietoikkuna.fi/?lang=en [↑](#footnote-ref-15)
16. https://kartkatalog.geonorge.no/ [↑](#footnote-ref-16)
17. https://www.worldpop.org/ [↑](#footnote-ref-17)
18. https://land.copernicus.eu/imagery-in-situ/eu-dem [↑](#footnote-ref-18)
19. https://kartkatalog.geonorge.no/ [↑](#footnote-ref-19)
20. Email from Joy Bhattacharjee (Oulu University) [↑](#footnote-ref-20)
