# Supplementary material for: Estimating societal benefits from Nordic catchments: An integrative approach using a final ecosystem services framework
Source: PLoS One. 2021 Jun 1;16(6):e0252352. doi: 10.1371/journal.pone.0252352 (PMC8168860; doi:10.1371/journal.pone.0252352)
Supplement: S3 File — (DOCX) [file pone.0252352.s003.docx]

Supplement 3 – Odense script for spatial quantification of TEV

Below we give sets of if-statements written in VBScript in ArcGIS 10.6, to be used in the field calculator in a vector dataset's attribute table.

Preparation

Create a fishnet vector dataset of 100x100m, clipped by the catchment boundary.

Spatial join land use (both CORINE and local) (center of cell)

Take the VP2 agriculture dataset, and add a column 'type', using:

*Dim output*

*if [AFGROEDE] = "Græs under 50% kløver/lucerne, meget lavt udbytte (omdrift)" then*

*output = "Grass"*

*elseif [AFGROEDE] = "Græs med kløver/lucerne, under 50 % bælgpl. (omdrift)" then*

*output = "Grass"*

*elseif [AFGROEDE] = "Græs og kløvergræs uden norm, over 50 % kløver (omdrift)" then*

*output = "Grass"*

*elseif [AFGROEDE] = "Græs og kløvergræs uden norm, under 50 % kløver (omdrift)" then*

*output = "Grass"*

*elseif [AFGROEDE] = "Græs til udegrise, omdrift" then*

*output = "Grass"*

*elseif [AFGROEDE] = "Græs til udegrise, permanent" then*

*output = "Grass"*

*elseif [AFGROEDE] = "Græs uden kløvergræs (omdrift)" then*

*output = "Grass"*

*elseif [AFGROEDE] = "Græs under 50% kløver/lucerne, ekstremt lavt udbytte (omdrift)" then*

*output = "Grass"*

*elseif [AFGROEDE] = "Græs under 50% kløver/lucerne, lavt udbytte (omdrift)" then*

*output = "Grass"*

*elseif [AFGROEDE] = "Miljøgræs MVJ-tilsagn (0 N), omdrift" then*

*output = "Grass"*

*elseif [AFGROEDE] = "Miljøgræs MVJ-tilsagn (0 N), permanent" then*

*output = "Grass"*

*elseif [AFGROEDE] = "Miljøgræs MVJ-tilsagn (80 N), omdrift" then*

*output = "Grass"*

*elseif [AFGROEDE] = "Udnyttet græs ved vandboring" then*

*output = "Grass"*

*elseif [AFGROEDE] = "Blandkorn, vårsået, helsæd" then*

*output = "Grains"*

*elseif [AFGROEDE] = "Grønkorn af vårbyg" then*

*output = "Grains"*

*elseif [AFGROEDE] = "Grønkorn af vårhavre" then*

*output = "Grains"*

*elseif [AFGROEDE] = "Grønkorn af vårrug" then*

*output = "Grains"*

*elseif [AFGROEDE] = "Grønkorn af vinterhvede" then*

*output = "Grains"*

*elseif [AFGROEDE] = "Grønkorn af vinterrug" then*

*output = "Grains"*

*elseif [AFGROEDE] = "Korn + bælgsæd under 50% bælgsæd" then*

*output = "Grains"*

*elseif [AFGROEDE] = "Korn og bælgsæd, helsæd, under 50% bælgsæd" then*

*output = "Grains"*

*elseif [AFGROEDE] = "Permanent græs og kløvergræs uden norm, over 50 % kløver" then*

*output = "Grass"*

*elseif [AFGROEDE] = "Permanent græs og kløvergræs uden norm, under 50 % kløver" then*

*output = "Grass"*

*elseif [AFGROEDE] = "Permanent græs ved vandboring" then*

*output = "Grass"*

*elseif [AFGROEDE] = "Permanent græs, lavt udbytte" then*

*output = "Grass"*

*elseif [AFGROEDE] = "Permanent græs, meget lavt udbytte" then*

*output = "Grass"*

*elseif [AFGROEDE] = "Permanent græs, normalt udbytte" then*

*output = "Grass"*

*elseif [AFGROEDE] = "Permanent græs, uden kløver" then*

*output = "Grass"*

*elseif [AFGROEDE] = "Permanent græs, under 50% kløver/lucerne" then*

*output = "Grass"*

*elseif [AFGROEDE] = "Permanent kløvergræs, over 50% kløver/lucerne" then*

*output = "Grass"*

*elseif [AFGROEDE] = "Lucernegræs, over 25% græs til slæt inkl. eget foder" then*

*output = "Grass"*

*elseif [AFGROEDE] = "Rajgræsfrø, alm." then*

*output = "Grains"*

*elseif [AFGROEDE] = "Rajgræsfrø, alm. 1. år, efterårsudlagt" then*

*output = "Grains"*

*elseif [AFGROEDE] = "Rajgræsfrø, ital." then*

*output = "Grains"*

*elseif [AFGROEDE] = "Vårbyg" then*

*output = "Grains"*

*elseif [AFGROEDE] = "Vårbyg, helsæd" then*

*output = "Grains"*

*elseif [AFGROEDE] = "Vårhavre" then*

*output = "Grains"*

*elseif [AFGROEDE] = "Vårhavre, helsæd" then*

*output = "Grains"*

*elseif [AFGROEDE] = "Vårhvede" then*

*output = "Grains"*

*elseif [AFGROEDE] = "Vårhvede, brødhvede" then*

*output = "Grains"*

*elseif [AFGROEDE] = "Vårhvede, helsæd" then*

*output = "Grains"*

*elseif [AFGROEDE] = "Vårrug" then*

*output = "Grains"*

*elseif [AFGROEDE] = "Vinterbyg" then*

*output = "Grains"*

*elseif [AFGROEDE] = "Vinterhvede" then*

*output = "Grains"*

*elseif [AFGROEDE] = "Vinterhvede, brødhvede" then*

*output = "Grains"*

*elseif [AFGROEDE] = "Vinterhvede, helsæd" then*

*output = "Grains"*

*elseif [AFGROEDE] = "Vinterhybridrug" then*

*output = "Grains"*

*elseif [AFGROEDE] = "Vinterrug" then*

*output = "Grains"*

*elseif [AFGROEDE] = "Vinterspelt" then*

*output = "Grains"*

*else output = "Other"*

*end if*

Spatial join this to ES cells using 'closest'. For Null cells and cells not under VP2 agriculture, reclassify as "Not agriculture".

Agriculture

For 'Type', turn those values not under VP2 agriculture into "Not agriculture", as well as Null values.

**ES_agriculture**

*dim output*

*if [type] = "Grains" then*

*output = 5500.00*

*elseif [Crop] = "Grass" then*

*output = 24676.00*

*elseif [Crop] = "Other" then*

*output = 28591.25*

*else output = 0*

*end if*

Correct for area (divide by ratio of area/10000) and correct for difference with spreadsheet

**Val_agriculture**

*dim output*

*if [Crop] = "Grains" then*

*output =* 233.23692

*elseif [Crop] = "Grass" then*

*output =* 1440.45481

*elseif [Crop] = "Other" then*

*output =* 157.05172

*else output = 0*

*end if*

Correct for difference with spreadsheet due to resolution effect of hectare cells.

Forestry

Spatial join agriculture field data to ES cells.

**ES_forestry**

*dim output*

*if [AFGROEDE] = "Skovdrift, alm." then*

*output = 445.5430*

*elseif [AFGROEDE] = "Bæredygtig skovdrift" then*

*output = 445.5430*

*elseif [AFGROEDE] = "Bæredygtig skovdrift i Natura 2000-område" then*

*output = 445.5430*

*else output = 0*

*end if*

Correct for area (divide by ratio of area/10000) and correct for difference with spreadsheet due to resolution effect of hectare cells.

**Val_forestry**

*[ES_forestr]** 30.3645074

Correct for difference with spreadsheet.

Game

**ES_game**

*dim output*

*if [Landuse] = "Dry nature" then*

*output = 0.7508*

*elseif [Landuse] = "Extensive agriculture" then*

*output = 0.7508*

*elseif [Landuse] = "Forest" then*

*output = 0.7508*

*elseif [Landuse] = "Intensive agriculture" then*

*output = 0.7508*

*elseif [Landuse] = "Unclassified agriculture" then*

*output = 0.7508*

*elseif [Landuse] = "Wet nature" then*

*output = 0.7508*

*else output = 0*

*end if*

Correct for area (divide by ratio of area/10000) and correct for difference with spreadsheet due to resolution effect of hectare cells.

**Val_game**

*[ES_game]* 2.217662267*

Peat

**ES_peat**

0

**Val_peat**

0

Hydropower

**ES_hydro**

0

**Val_hydro**

0

Foraging

**ES_foraging**

0

**Val_foraging**

0

Water consumption

**ES_water**

*Dim output*

*If [Landuse] = "Sea" then*

*Output = 0*

*Else output = 164130.6682*( [Shape_Area]/10000)*

*End if*

Correct for difference with spreadsheet due to resolution effect of hectare cells.

**Val_water**

*0.000628104 * [ES_water]*

Recreational hunting

**ES_hunting**

*dim output*

*if [ES_game] > 0 then*

*output = 0.0887*

*else output = 0*

*end if*

*Correct for area (divide by ratio of area/10000) and correct for difference with spreadsheet due to resolution effect of hectare cells.*

**Val_hunting**

*65*[ES_hunting]*

Recreational fishing

Add column fishingwater. Select by location where cells intersect the fishingwater layer. Set to 1 in that case, 0 otherwise.

**ES_fishing**

*dim output*

*if [Fishingwater] = 1 then*

*output = (3.64755* [Shape_Area]/10000)*

*else output = 0*

*end if*

Correct for difference with spreadsheet due to resolution effect of hectare cells.

**Va_fishing**

*[ES_fishing]*186.0062814*

Recreation day trips

Converted SSB population polygons to random points within cell, using Create Random Points. Then used point density with a 7km search radius to create a new recreation raster of 100x100m (check the point project). Create points from raster. Then join these values to the ES polygon cells using Spatial Join with mean value and a buffer of 50m.

Add an attribute for:

- Distance to road
- Distance to water

For SDI:

Add to ES cells a new attribute, copying ObjectID and call it VP2split. Go to the advanced editor and split the AR50 layer using these cells. Explode the multipart features. Spatial join the VP2split attribute (have their center in). Export that VP2 layer to Excel. Make a pivot table over VP2split and Landuse, with both as rows. Take the percentage of parent row for Landuse, and copy that to a normal table. Mess around with that until you get a column Hacell with which AR50split cell the Landuse belongs to, and a column with an SDI per cell of MINUS (Landcoverfraction)*ln(Landcoverfraction). Then create a pivotable out of that using Hacell and SDI, and take the sum per Hacell to find actual SDI. Save that as a separate excel file. Add that to the ES cells in GIS using the Join Field tool.

Then for water distance, road distance and SDI each add a score using the histogram functions (take a X Y scatterplot in Excel for the correct function), giving you 3 new columns. Where SDI gives no value, give it a score of 0.

Add a new column, Rec_score. Calculate this by taking:
gridcode*((waterscore/50)+(roadscore/50)+(SDIscore/50))*(shape area/10000)

If Landuse is built up area, set the Rec_score to 0.

Then convert these values to fraction of recreating population:

[Rec_score]/(sum of recscore/total recreants)

Then add ES_recdays = [Recreants]*average recdays

Then add Val_recdays = [Recreants]*average value per recreant

Correct for difference with spreadsheet due to resolution effect of hectare cells.

Carbon sequestration

**ES_carbon**

*dim output*

*if [Landuse] = "Forest" then*

*output = (0.575*( [Shape_Area]/10000)*

*elseif [Landuse] = "Lake" then*

*output = (0.185*( [Shape_Area]/10000)*

*else output = 0*

*end if*

Correct for difference with spreadsheet due to resolution effect of hectare cells.

**Val_carbon***[ES_carbon]*3.67*64.46043165*

Flood prevention

- Create fishnet 25m2
- Link fishnet to flood area (select by location with ‘have their center in’ and extract)
- Clip to catchment boundary if cells overflow
- Link fishnet to CORINE (spatial join with ‘have their center in’)
- Delete all cells in waterways and lakes
- Add attribute ‘Downstream’
- Make a layer of all waterways with high river order
- Select all flood risk cells within an area of those large, depending on the width of floodplain, and define ‘Downstream’ as 1. For the rest define ‘Downstream as 0

| **Catchment** | **Downstream river order** | **Buffer around downstream waterway** |
| --- | --- | --- |
| Haldenvassdraget | >4 | 1.000m |
| Orrevassdraget | >2 | 500m |
| Odense | >2  + highest order for separate streams going into the fjord | 500m |
| Simojoki | >4 | 1000m |
| Sävjaån | Separate method, see below |  |
| Vindelälven | No river order data, took central stream only | 1000m, manually removed some side streams |

- Add attribute ‘Value’ and add numbers from De Moel et al (see script on next page)

**Pre-logic script code:**

*dim output*

*if [Code_18] = "111" then*

*output = 157500*

*elseif [Code_18] = "112" then*

*output = 157500*

*elseif [Code_18] = "121" then*

*output = 163750*

*elseif [Code_18] = "123" then*

*output = 163750*

*elseif [Code_18] = "124" then*

*output = 163750*

*elseif [Code_18] = "131" then*

*output = 163750*

*elseif [Code_18] = "141" then*

*output = 157500*

*elseif [Code_18] = "142" then*

*output = 157500*

*elseif [Code_18] = "211" then*

*output = 4375*

*elseif [Code_18] = "222" then*

*output = 4375*

*elseif [Code_18] = "231" then*

*output = 4375*

*elseif [Code_18] = "242" then*

*output = 4375*

*elseif [Code_18] = "243" then*

*output = 4375*

*elseif [Code_18] = "311" then*

*output = 625*

*elseif [Code_18] = "312" then*

*output = 625*

*elseif [Code_18] = "313" then*

*output = 625*

*elseif [Code_18] = "322" then*

*output = 625*

*elseif [Code_18] = "324" then*

*output = 625*

*elseif [Code_18] = "411" then*

*output = 0*

*elseif [Code_18] = "412" then*

*output = 0*

*elseif [Code_18] = "421" then*

*output = 0*

*else output = 0*

*end if*

**How to calculate prevented flood area**

- Start ‘zonal statistics as table’: link mean DEM values to floodedcells features using OBJECTID.
- Start ‘join field’ to link the new table to the floodedcells using OBJECTID.
- Add ‘edge’ attribute’
- Dissolve floodedcells on join count. Convert that to a polyline ‘floodedge’.
- Select by location on flooded cells: where it intersects floodedge. Define ‘edge’ attribute there as 1. For the others set to 0.
- Select the edge cells, export them. Perform a spatial join between floodedcells and those edge cells, using CLOSEST GEODESIC.
- Add ‘flooddepth’ attribute
- Calculate flood depth per cell:

*dim output*

*if [Edge] = "1" then*

*output = 0.1*

*else output = [Edge_DEM] - [Cell_DEM] +0.1*

*end if*

- Transform negative values to 0.1.
- Add ‘floodvolume’ attribute
- Calculate flood volume per cell by multiplying depth with surface area
- Export to Excel and calculate upstream damage, avoided downstream damage and net benefit

**How to calculate prevented flood depth**

- Calculate current downstream flooded area and damage
- Divide upstream volume by the sum of current downstream area and avoided downstream area: this yields extra volume per area
- Create a new column in the spreadsheet adding this volume to the current downstream volume (times 625 to convert to cell size)
- Create a new column in the spreadsheet converting to new depth
- Create a new column in the spreadsheet converting to new damage
- Subtract old from new

Create upstream dataset (of 25m2 cells). Spatial join ES cells that have their centroid in the upstream features, with a search distance of 5 m, including the SUM of flood volume.

Add ES_flooding and put in Floodvolume values. Change NULL to zero.

Correct for area (divide by ratio of area/10000) and correct for difference with spreadsheet due to resolution effect of hectare cells.

Add Val_flooding and multiply Floodvolume with value per volume

Stakeholders

**Land owner**

*dim output*

*if [TEV] > 0 then*

*output = ( [Val_agricu]+ [Val_forest]+ [Val_flooding])/ [TEV]*

*else output = 0*

*end if*

**Visitor**

*dim output*

*if [TEV] > 0 then*

*output = ([Val_game]+ [Val_huntin]+ [Val_fishin] + [Val_recday])/[TEV]*

*else output = 0*

*end if*

**Large extractor**

*dim output*

*if [TEV] > 0 then*

*output= ([Val_water])/[TEV]*

*else output = 0*

*end if*

**Global society**

*dim output*

*if [TEV] > 0 then*

*output = [Val_carbon]/ [TEV]*

*else output = 0*

*end if*

**Mainuser**

*dim output*

*if [Landowner] > [Visitor] AND [Landowner] >[Globalsociety] AND [Landowner] > [Resident] then*

*output = "Landowner"*

*elseif [Visitor] > [Globalsociety] AND [Visitor] > [Resident] then*

*output = "Visitor"*

*elseif [Resident] > [Globalsociety] then*

*output = "Resident"*

*elseif [TEV] = 0 then*

*output = "No one"*

*else output = "Global society"*

*end if*

THEN:

Create two new layers from land use: Forest and Agriculture.

Add two new attributes to ES_subcatchment: Forestclose and Agricultureclose. Select by location with 500m radius on the two new layers and set to 1 when match.

Add two new attributes to ES_subcatchment: Fortoagri and agritofor. Select by attributes where they might shift, based on closeness of the other and soil type, and set to 1 when appropriate.

Export to excel. Calculate effects per stakeholder group for each shift.
